# Supplementary material for: Protein nonadditive expression and solubility contribute to heterosis in Arabidopsis hybrids and allotetraploids
Source: Front Plant Sci. 2023 Sep 14;14:1252564. doi: 10.3389/fpls.2023.1252564 (PMC10538547; doi:10.3389/fpls.2023.1252564)
Supplement: Supplementary file 1 [file DataSheet_1.pdf]

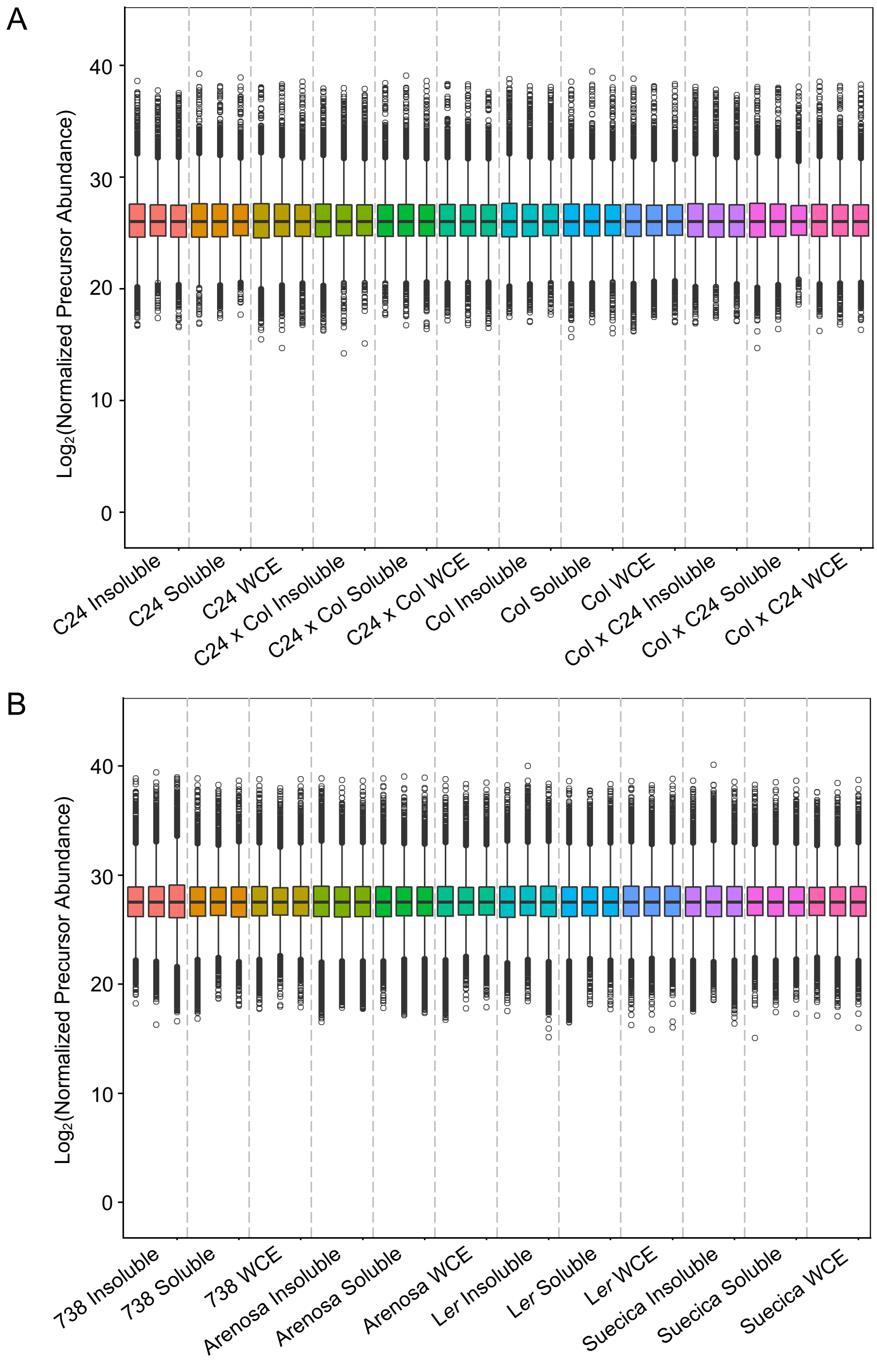

Supplementary Fig. 1. Log_2(normalized precursor abundance) values of the peptides among all samples analyzed. Precursor ion abundance is calculated based on the chromatogram peak area of the precursor ion. (A) Log_2(normalized precursor abundance) of all peptides identified in the intraspecific hybrid samples (B) Log_2(normalized precursor abundance) of all peptides identified in the interspecific polyploid samples.

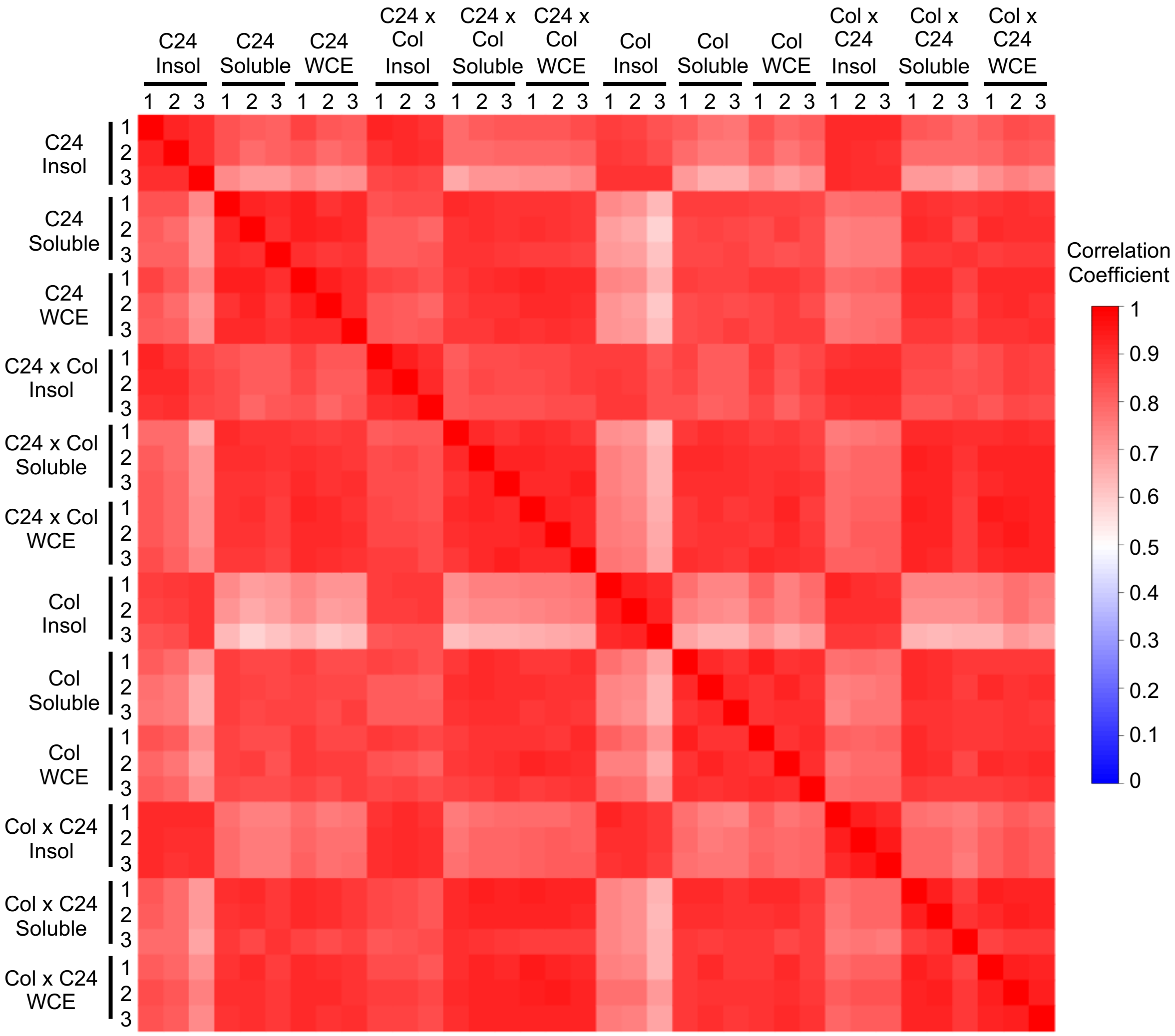

Supplementary Fig. 2. Reproducibility of protein abundances in intraspecific hybrids. Pearson correlation of protein abundances for all intraspecific hybrid samples. There is a high degree of reproducibility between biological replicates, with the insoluble fractions showing lower levels of correlation with soluble and whole cell extract fractions.

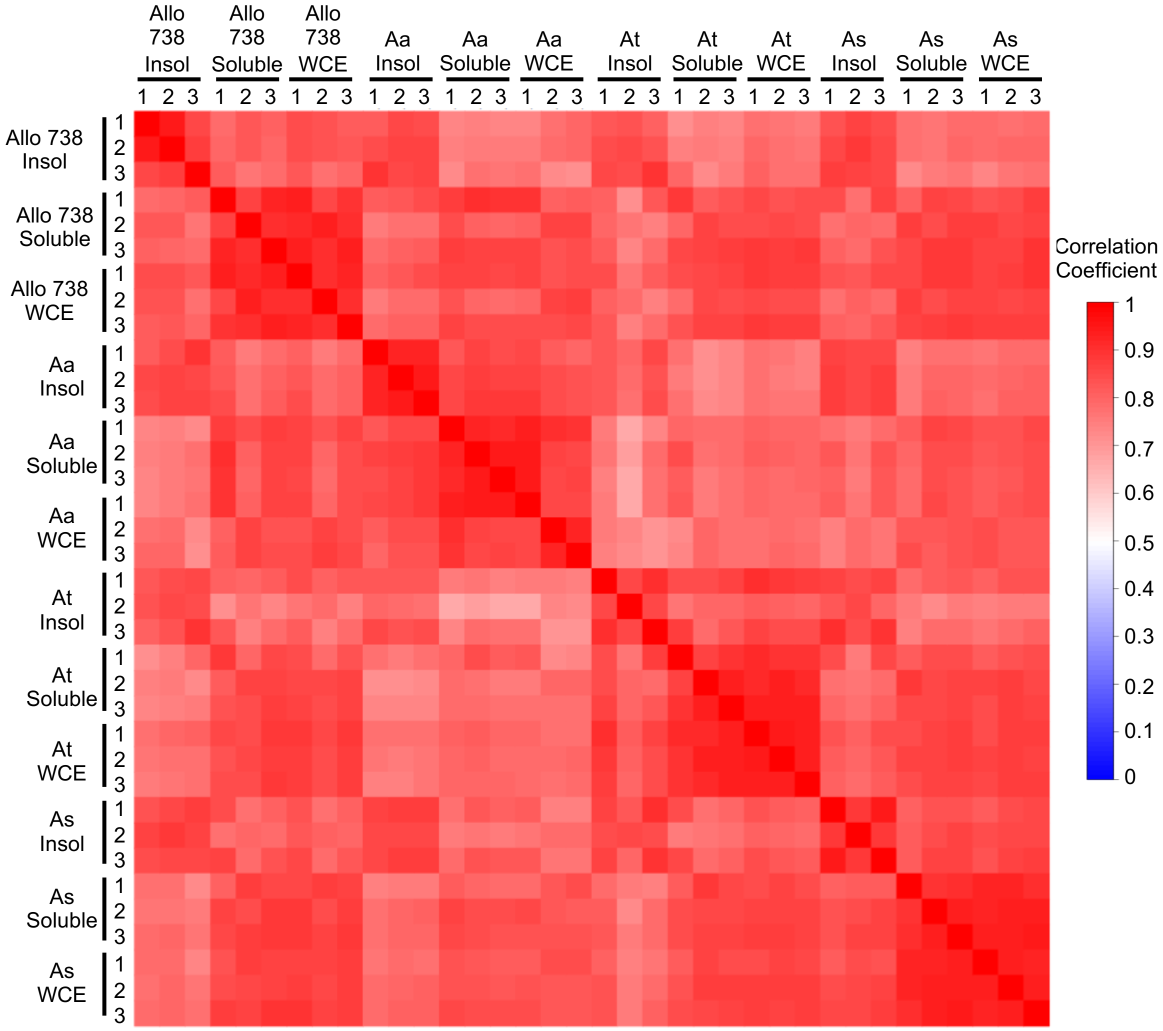

Supplementary Fig. 3. Reproducibility of protein abundance in allotetraploids. Pearson correlation of protein abundances for all allotetraploid samples. There is a high degree of reproducibility between biological replicates, with the insoluble fractions showing lower levels of correlation with soluble and whole cell extract fractions.

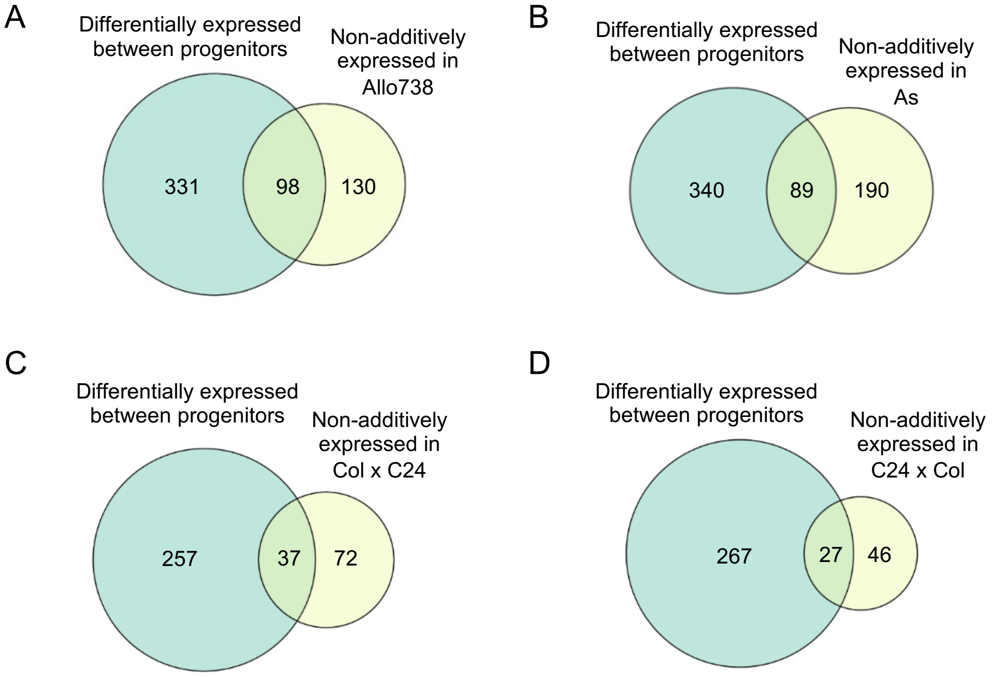

Supplementary Fig. 4. Overlap of the proteins that were differentially expressed between the parents with the non-additively expressed proteins. The proteins differentially expressed between the progenitors of each hybrid or allotetraploid were overlapped with the non-additively expressed proteins in (A) All738, (B) *A. suecica*, (C) F_1 (Col x C24), and (D) F_1 (C24 x Col).

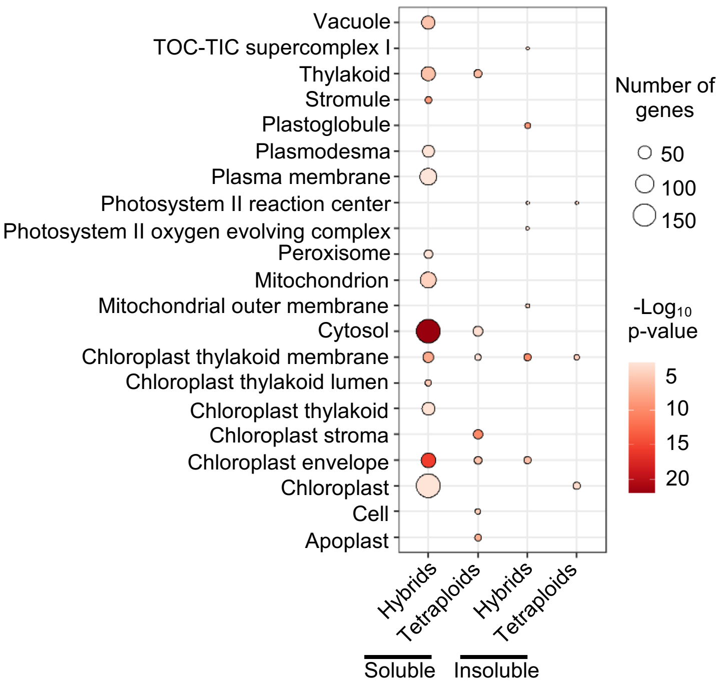

Supplementary Fig. 5. Enrichment of soluble fractions in cytosolic proteins and of insoluble fractions in proteins localized to membranes. GO cellular component enrichment of proteins significantly enriched in the insoluble and soluble fractions in both experiments. The soluble fraction shows strong enrichment across several cellular components including the cytosol, whereas the insoluble fraction only shows enrichment for chloroplast proteins, particularly those in the thylakoid membrane.

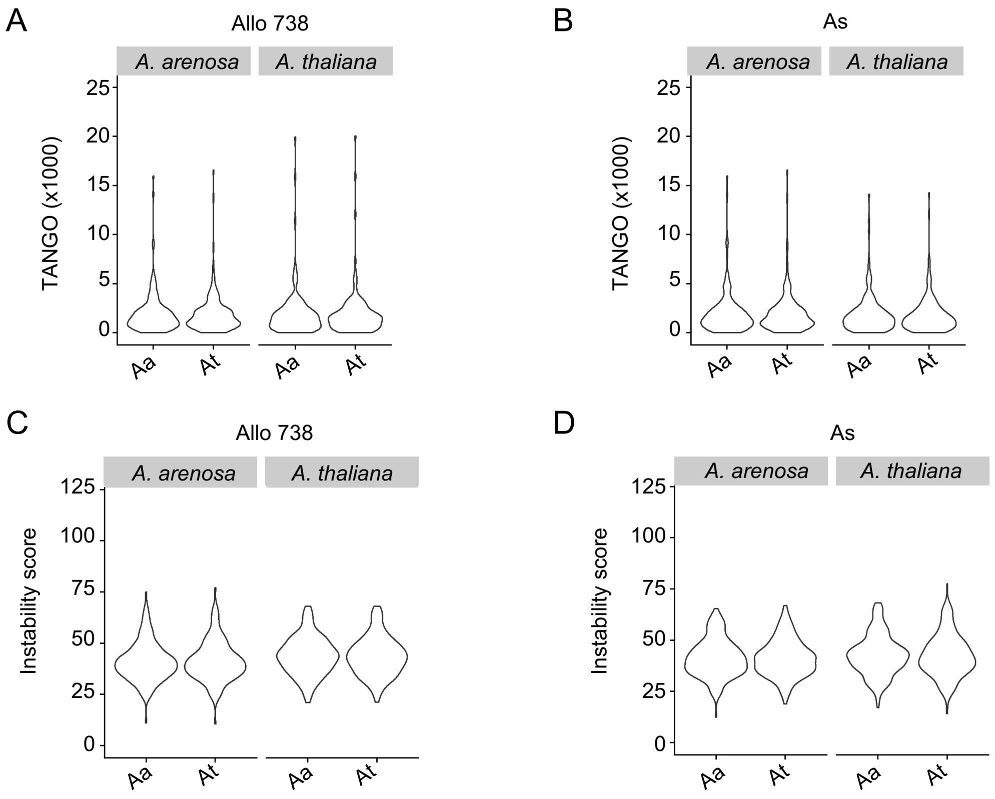

Supplementary Fig. 6. Evaluation of homoeolog-specific expression found no obvious homoeolog-specific expression of more soluble proteins in *Arabidopsis* allotetraploids. The distribution of TANGO scores of the *A. thaliana* and *A. arenosa* homoeologs of all proteins that display homoeolog biased expression in (A) Allo738 and (B) *A. suecica* and the distribution of instability scores of the *A. thaliana* and *A. arenosa* homoeologs of all proteins that display homoeolog biased expression in (C) Allo738 and (D) *A. suecica*. For each graph, grey boxes divide graph into proteins that displayed either *A. thaliana* or *A. arenosa* subgenome bias, and the two distributions within these panels indicate the distribution of TANGO or instability scores for the *A. arenosa* and *A. thaliana* homoeologs of the proteins that display this biased expression.

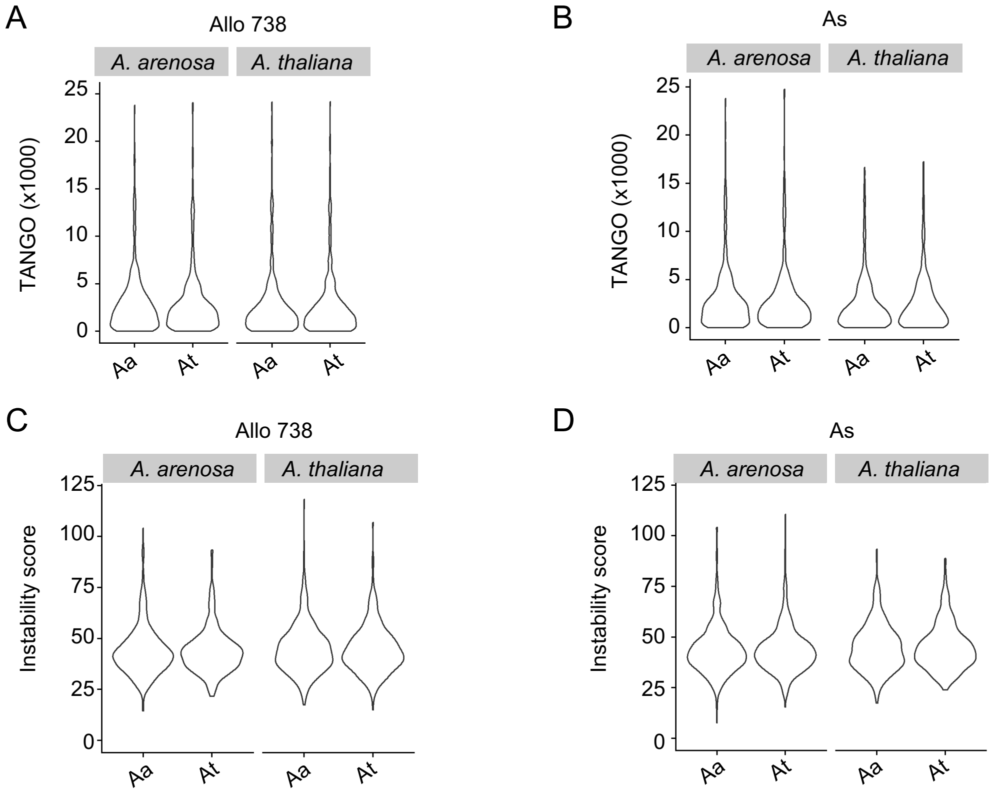

Supplementary Fig. 7. Evaluation of homoeolog-specific expression found no obvious homoeolog-specific expression of more soluble proteins in *Arabidopsis* allotetraploids. The distribution of TANGO scores of the *A. thaliana* and *A. arenosa* homoeologs of all proteins that display homoeolog biased expression in (A) Allo738 and (B) *A. suecica* and the distribution of instability scores of the *A. thaliana* and *A. arenosa* homoeologs of all proteins that display homoeolog biased expression in (C) Allo738 and (D) *A. suecica*. For each graph, grey boxes divide graph into proteins that displayed either *A. thaliana* or *A. arenosa* subgenome bias, and the two distributions within these panels indicate the distribution of TANGO or instability scores for the *A. arenosa* and *A. thaliana* homoeologs of the proteins that display this biased expression.
